# Antimicrobial Activity and Crystallization Features in Bio-Based Composites of PLLA and MCM-41 Particles Either Pristine or Functionalized with Confined Ag Nanowires

**DOI:** 10.3390/polym15092084

**Published:** 2023-04-27

**Authors:** Tamara M. Díez-Rodríguez, Enrique Blázquez-Blázquez, Marta Fernández-García, Alexandra Muñoz-Bonilla, Ernesto Pérez, María L. Cerrada

**Affiliations:** Instituto de Ciencia y Tecnología de Polímeros (ICTP-CSIC), Juan de la Cierva 3, 28006 Madrid, Spain; t.diez@ictp.csic.es (T.M.D.-R.); enrique.blazquez@ictp.csic.es (E.B.-B.); martafg@ictp.csic.es (M.F.-G.); sbonilla@ictp.csic.es (A.M.-B.);

**Keywords:** PLLA, neat and hybrid MCM-41 silica, Ag nanowires, nucleating and reinforcing effect, microbes, antimicrobial properties

## Abstract

Composites based on an *L*-rich poly(lactic acid) (PLLA) and MCM-41, either neat or modified with a silver (MCM-41@Ag), are achieved by solvent casting, being next processed by compression molding. Ag is mainly embedded as nanowires within the hybrid MCM-41@Ag particles, enabling its antimicrobial character. In these composites, the PLLA thermal stability, nucleation efficiency, and mechanical response are dependent on the MCM-41 nature and, to a lesser extent, on its content. Thus, differences in transitions of the PLLA matrix are noticed during cooling at 10 °C/min and in the subsequent heating when composites with neat or modified MCM-41 are compared. A very remarkable nucleation effect is played by pristine MCM-41, being inferior when MCM-41@Ag is incorporated into the PLLA. Wide angle X-ray scattering (WAXS) measurements using synchrotron radiation and performed under variable-temperature conditions in the composites containing MCM-41@Ag indicate that during cold crystallization, the disordered α′ polymorph is initially formed, but it rapidly transforms into ordered α crystals. A long spacing peak, clearly seen in pure PLLA, appears as a small shoulder in PLLAMCM@Ag4 and is undetectable in PLLAMCM@Ag9 and PLLAMCM@Ag20. Furthermore, an increase in MH with the silica content is found in the two sets of composites, the higher MH values being observed in the family of PLLA and MCM-41@Ag. Finally, remarkable antimicrobial features are noticeable in the composites with MCM-41@Ag since this modified silica transfers its biocidal characteristics into the PLLA composites.

## 1. Introduction

Ordered mesoporous silicas show great potential for attaining polymeric based (nano)composites. Among them, the Mobil Crystalline Materials (MCM) were obtained in 1992 by Mobil [1], becoming the MCM-41 its most known member. By changing the type of surfactant used during the synthetic protocol, another mesoporous silica family was achieved at Santa Barbara University of California in 1998 [2]. The SBA-15 is its best exponent. Both MCM-41 and SBA-15 are characterized as being constituted of parallel one-dimensional pores disposed of in a hexagonal morphology. The pore diameter is their main difference, which is about 3 nm in the former and ranges from 5 to 10 nm in SBA-15. These mesoporous particles can act either as catalyst carriers or as reinforcement, if their incorporation is performed by in situ polymerization [3,4] or only as a filler if melt compounding protocols, such as extrusion, are used [5,6].

This reinforcing effect could be further maximized if the confinement of the polymeric chains exists within the empty pores of mesoporous particles [7]. Changes in the main (100) reflection of the hexagonal arrangements for SBA-15 or MCM-41, proved through real-time variable-temperature X-ray diffraction experiments with synchrotron radiation at small angles (SAXS), have been a decisive sensor in different semicrystalline nanocomposites for elucidating existence of confinement [8,9]. In pristine silicas, the (100) diffraction (position, intensity, or width) is not altered with temperature in the 20 °C to 200 °C interval. On the contrary, materials composed of polymer and mesoporous silica display an evident discontinuity in the intensity of this (100) diffraction. Confinement was hence detected in nanocomposites based on isotactic polypropylene and poly(ε-caprolactone) [7,10], being corroborated also through DSC by the presence of a secondary endothermic event located at temperatures below the main melting process. Nevertheless, no evidence of confined crystalline macrochains has been noted in hybrids of *L*-rich poly(lactic acid)s (PLLA) and SBA-15 silica, a fact attributed to the much more reduced crystallization capacity, which is inherent in PLLA, along with its slower rate compared with those features exhibited by the aforementioned polymers.

These two just-mentioned adverse characteristics of the PLLA have been improved by the addition of neat SBA-15 particles since they have been found to play a nucleating role. The influence of silica incorporation is dependent on the amount of SBA-15 added [6,11], becoming triggered with increasing content, and on the fraction of *D* isomer present in the PLLA matrix [12]. As usual, isothermal crystallization from the glassy state is noticeably faster than that from the molten state, and the SBA-15 nucleating effect was found to be more significant when crystallization started from the melt [11]. Moreover, an atypical crystallization window was noted for the development of the α′ and α polymorphs in a PLLA with about 4% in *D* stereoisomer content and in its extruded composites with SBA-15. Thus, the α′ crystals required crystallization temperatures below 80 °C and long times, while the α modification, which is the most common and stable polymorph, was the only polymorph obtained above 90 °C [6]. Formation of α′ and α forms were previously described to be developed at annealing temperatures below around 100 °C and above 120 °C, respectively, with both crystalline cells coexisting in the temperature range from 100 to 120 °C [13,14,15,16,17,18].

Another very remarkable feature exhibited by mesoporous silicas is their easy chemical modification, enabling the incorporation of numerous functionalities. Protection against all types of microbes (bacteria, fungi, viruses, etcetera) has turned out to be a great challenge as of late and even more so after the SARS-CoV-19 worldwide pandemic. Several chemical and physical methods have been developed over the years to eradicate bacteria and other toxic germs. The most extreme approach is sterilization, which involves the full removal of vegetative cells, endospores, and viruses from the target [19]. This methodology is mainly applied to medical, manufacturing, laboratory, and food industry settings. Sterilization can be achieved either by physical means, such as applying heat, pressure, or radiation as well as filtering through an appropriate membrane, or by chemical means through the action of sterilants, which are effective in killing all microbes and viruses and can also eliminate endospores with appropriate exposure time. Nevertheless, sterilization requires protocols that are not practical or necessary in many settings. Various other methods are used in clinical and nonclinical situations to reduce the microbial load on items: disinfection, sanitization, antisepsis, or degerming [19]. In addition, the development of new types of materials can help to eradicate microorganisms. These are mainly divided into three wide categories:  (a) synthetic polymers that mimic naturally occurring bactericidal molecules/peptides; (b) microbe-repelling anti-adherence polymers; (c) inorganic/organic materials with a slow releasing of biocides, such as heavy metals or oxides (primarily based on silver), small molecule biocides, halogen species, or nitric oxide [20]. The beneficial capability of silver (Ag) for interacting with harmful substances, provoking their inactivation, is known from ancient times. Silver nanoparticles (Ag NPs) exert their antibacterial effects at several levels since they act in the bacterial wall and by blocking electron transfer, as well as in the cell respiration and replication of damaging proteins, RNA, and DNA [21]. On the other hand, metal oxides can eradicate bacteria via electrostatic interactions, changing the integrity of bacterial membranes and the generation of free radicals, which are toxic to bacteria. Higher flexibility can be attained by Ag/metal oxide combination in a single material, being of growing interest to learn the ability to eradicate microorganisms of these systems, such as Ag-TiO_2_ [22] and Ag-SnO_2_ [23].

Thus, Ag NPs have become the focus of intensive research due to their application as antimicrobials and in other fields, such as catalysis, optics, or biomaterial production [24]. However, their practical applicability is reduced in the case of the formation of Ag NPs aggregates. Mesoporous silicas can solve this drawback since they can act as carriers, leading to hybrid particles based on them and Ag.

The aim of this investigation is focused on the preparation of composites based on PLLA and hybrid particles of MCM-41 functionalized with Ag (MCM-41@Ag) in order to compare, on the one hand, the effect that MCM-41 decoration plays in the PLLA crystallization and, on the other hand, to achieve PLLA composites with biocidal characteristics. The former requires the obtainment of both types of PLLA composites, i.e., from either neat or from hybrid Ag mesoporous particles. Different approaches can be found in the literature, being selected one that allows achieving Ag nanowires within the mesoporous channels [25]. Once the characterization of these modified silicas is carried out, evaluation for the several phase transitions and the crystalline phases developed will be performed. Furthermore, the preliminary analysis of the mechanical response in all the resultant composites will also be carried out. Finally, the study of the antimicrobial characteristics against bacteria (Gram-negative and Gram-positive) and fungus is accomplished in those hybrids containing Ag. All these studies will involve the utilization of numerous techniques, including transmission electron microscopy (TEM), scanning electron microscopy (SEM), thermogravimetry (TGA), differential scanning calorimetry (DSC), X-ray scattering experiments with synchrotron radiation at wide (WAXS) or small angles (SAXS), microhardness tests and antimicrobial measurements.

## 2. Materials and Methods

### 2.1. Materials and Chemicals

A commercially available L-rich polylactide (PLLA) from NatureWorks^®^ (Minnetonka, MN, USA) (labeled as Ingeo™ Biopolymer 6202D, with a density of 1.24 g/cm^3^ and a content in L-isomer units of about 98 mol%) is used in this study. Its weight-average molecular weight (M_w_) and molecular weight dispersity are 118,600 g/mol and 1.6, respectively, as determined by gel permeation chromatography (GPC) [12]. The pristine MCM-41 particles were purchased from Sigma-Aldrich (San Luis, MO, USA) (specific surface area, SBET = 966 m^2^/g; average mesopore diameter, Dp = 2.9 nm [26]) and were used as received. Chloroform (99.0–99.4%, Sigma-Aldrich, San Luis, MO, USA) was used as a solvent for the casting processing. The AgNO3, BioXtra grade, was purchased from Sigma-Aldrich, and it was employed as received.

Antimicrobial tests required the following chemicals and organisms: sodium chloride solution (NaCl suitable for cell culture, BioXtra) and phosphate-buffered saline powder (PBS, pH 7.4) both from Sigma-Aldrich (San Luis, MO, USA). Columbia agar plates (Sheep blood (5%)) were purchased from Deltalab (Barcelona, Spain), and BBL Mueller–Hinton broth was purchased from Becton, Dickinson, and Company (Madrid, Spain), which was used as a microbial growth medium. American Type Culture Collection (ATCC): *Pseudomonas aeruginosa* (*P. aeruginosa*, ATCC 27853), *Escherichia coli* (*E. coli*, ATCC 25922), *Staphylococcus epidermidis* (*S. epidermidis*, ATCC 12228) and *Staphylococcus aureus* (*S. aureus*, ATCC 29213) were used as bacterial strains and yeast *Candida parapsilosis* (*C. parapsilosis* ATCC 22109) was used as a fungal strain, being all of them purchased from Oxoid (Hampshire, UK).

### 2.2. Preparation of Hybrid MCM-41@Ag Particles

Functionalization of MCM-14 particles was performed following the protocol previously described in the literature [25] and recently used for modification of SBA-15 particles [27]. It briefly consisted: of 500 mg of MCM-14 powder soaked in 250 mL of 0.2 M AgNO_3_ EtOH:H_2_O (1:1 *v*/*v*) solution, and the suspension was stirred overnight at room temperature. The product was filtered off, rinsed with deionized water, and subjected to thermal treatment at 300 °C in the air for 2 h to decompose the impregnated AgNO_3_, enabling reduction from Ag^+^ cations to metallic Ag^0^. The sample impregnated with AgNO_3_ was colorless, while a grey color appeared once the sample was treated at 300 °C.

### 2.3. Composites Preparation

**Scheme 1:**
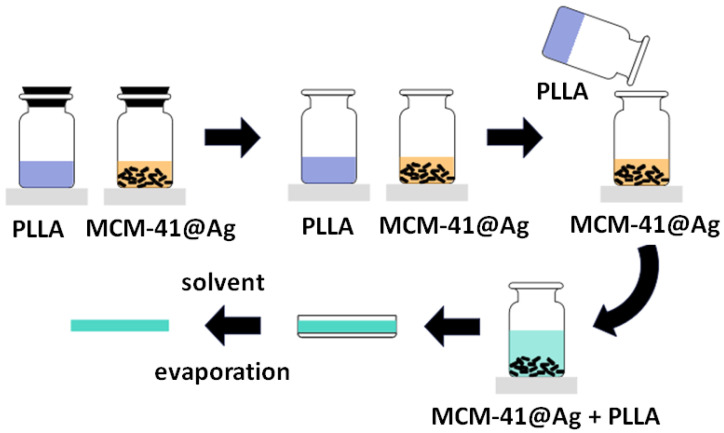
Protocol for the obtainment of PLLAMCM@Ag composites specified for the hybrid MCM-41@Ag particles. The PLLAMCM materials are prepared analogously using pristine MCM-41 silica.

Composites with different contents in either pristine MCM-41 or hybrid MCM-41@Ag particles were achieved as follows (see Scheme 1): an appropriate amount of pure MCM-41 or decorated MCM-41@Ag silica was dispersed in chloroform at the same time that a PLLA/chloroform solution (6 wt.% in PLLA) was prepared. Both dispersion and solution were stirred for 18 h at room temperature. Afterward, a dispersion containing neat or modified MCM-41 particles was added to the PLLA/chloroform solution, and this PLLA/silica/chloroform dispersion was additionally stirred for 6 h at room temperature before they were poured into Petri dishes and dried at room temperature for 48 h. The resultant composite films were additionally dried in a vacuum oven at 85 °C for 2 h.

### 2.4. Obtainment of Films

These materials were subsequently processed by compression molding in a hot-plate Collin press. Initially, the material was maintained at a temperature of 195 °C and at a pressure of 30 bar for 6 min. Afterward, a cooling process at a relatively rapid rate of around 80 °C/min and at a pressure of 30 bar was applied to the different composites from their molten state to room temperature. These original compression-molded films were totally amorphous, as shown below.

Films of composites prepared using pure MCM-41 particles contain an amount in mesoporous silica of 2, 4, and 9 wt.% as determined by TGA, and they are named PLLAMCM2, PLLAMCM4, and PLLAMCM9, respectively. The composition of those materials incorporating hybrid MCM-41@Ag particles has been slightly changed in order to include a significant content in Ag to provide them with biocidal characteristics. Thus, the amount in hybrid particles (silica plus Ag) is 4, 9, and 20 wt.%, being these compounds designated as PLLAMCM@Ag4, PLLAMCM@Ag9 and PLLAMCM@Ag20, respectively.

### 2.5. Transmission Electron Microscopy

Morphological details of the mesoporous MCM-41, either pristine or decorated with Ag, were obtained by transmission electron microscopy (TEM). Measurements were performed at room temperature in a 200 kV JEM-2100 JEOL microscope (JEOL Ltd., Tokyo, Japan). The particles were dispersed in acetone in an ultrasonic bath for 5 min and then deposited in a holder prior to observation.

### 2.6. Scanning Electron Microscopy

Experiments of high-resolution field emission scanning electron microscopy (FESEM) were carried out in an S-8000 Hitachi equipment (Hitachi High-Tech Corporation, Tokyo, Japan) at room temperature in different cryo-fractured sections of composites at distinct mesoporous content. Those thin sections of around 30 nm were cut by cryo-ultramicrotomy (Leica EM UC6, Leica Microsystems GmbH, Wetzlar, Germany) at −120 °C and deposited in a square mesh copper gride (CF-400-Cu, Ted Tella Inc., Redding, CA, USA). Furthermore, chemical composition analysis was carried out by coupling a dispersive energy X-ray (EDX) device into the microscope, allowing estimation of the amount of Ag in the MCM-41@Ag particles.

### 2.7. X-ray Experiments with Conventional and Synchrotron Radiation

Wide angle X-ray Diffraction (WAXD) patterns were recorded for characterizing neat and functionalized MCM-41 particles, at room temperature in the reflection mode, by using a Bruker D8 Advance diffractometer provided with a PSD Vantec detector (from Bruker, Madison, WI, USA). Cu Kα radiation (λ = 0.15418 nm) was used, operating at 40 kV and 40 mA. The parallel beam optics was adjusted by a parabolic Göbel mirror with horizontal grazing incidence Soller slit of 0.12° and LiF monochromator. The equipment was calibrated with different standards. A step scanning mode was employed for the detector. The diffraction scans were collected with a 2θ step of 0.024° and 0.2 s per step.

Real-time variable-temperature simultaneous SAXS/WAXS experiments were carried out with synchrotron radiation in beamline BL11-NCD-SWEET at ALBA (Cerdanyola del Vallès, Barcelona, Spain) at a fixed wavelength of 0.1 nm. A Pilatus detector has been used for SAXS (off beam, at a distance of 296 cm from the sample) and a Rayonix one for WAXS (at about 14.6 cm from the sample and a tilt angle of around 29 degrees). A Linkam Unit, connected to a cooling system of liquid nitrogen, was employed for the temperature control. The calibration of spacings was obtained by means of silver behenate and Cr_2_O_3_ standards. The initial 2D X-ray images were converted into 1D diffractograms as a function of the inverse scattering vector, *s* = 1/*d* = 2 sin θ/λ, by means of pyFAI python code (ESRF), modified by ALBA beamline staff. Film samples of around 5 × 5 × 0.1 mm were used in the synchrotron analysis.

### 2.8. Thermogravimetric Analysis

Thermogravimetric analysis (TGA) was performed in a Q500 equipment of TA Instruments (New Castle, DE, USA), under nitrogen or air atmosphere at a heating rate of 10 °C/min. Degradation temperatures of the distinct materials are determined, as well as the exact SBA-15 amount incorporated into the composites prepared by extrusion, which has been estimated as an average of values obtained from the two environments.

### 2.9. Differential Scanning Calorimetry

Calorimetric analyses were carried out in a TA Instruments Q100 calorimeter connected to a cooling system and calibrated with different standards. The sample weights were around 3 mg. A temperature interval from −30 to 180 °C was studied at a heating rate of 10 °C/min. For the determination of crystallinity, a value of 93.1 J/g was used as the enthalpy of fusion of a perfectly crystalline material [28,29].

### 2.10. Microhardness

A Vickers indentor attached to a Leitz microhardness tester was used to perform microindentation measurements undertaken at 23 °C. A contact load of 0.98 N and a time of 25 s were employed. Microhardness, MH, value (in MPa) was calculated according to the relationship [30,31]:

(1) $$MH=2\sin68^{\circ }\left(\frac{P}{d^{2}}\right)$$

where P (in N) is the contact load and d (in mm) is the diagonal length of the projected indentation area. Diagonals were measured in the reflected light mode within 30 s of load removal using a digital eyepiece equipped with a Leitz computer counter printer (RZA-DO).

### 2.11. Antimicrobial Tests

#### 2.11.1. Minimum Inhibitory Concentration (MIC) of Pristine and Functionalized with Ag Mesoporous Silica

The minimum inhibitory concentration (MIC) values were determined following a standard broth dilution method according to the Clinical Laboratory Standards Institute (CLSI) [32]. Bacterial cells were cultured on a 5% sheep blood Columbia agar plate for 24 h at 37 °C. The concentration of bacterial suspension was adjusted with saline solution with a concentration of ~10^8^ colony-forming units (CFU) mL^−1^ (turbidity equivalent to ca. 0.5 McFarland turbidity standard). Subsequently, the suspension was diluted with fresh Mueller-Hinton broth to ~10^6^ CFU mL^−1^. The bacterial suspension was then mixed with several concentrations of freshly prepared mesoporous silica solution by serial dilutions in a 96-well plate. This mesoporous silica solution was prepared in Muller-Hilton broth medium at a concentration of 1000 μg mL^−1^. Then, 100 μL from each silica solution was added in the first column of a 96-well microplate, while 50 μL of fresh broth was added into the rest of the wells. Serial dilutions were performed from the first column, followed by the addition of 50 μL of the bacterial suspension in each well to yield a total volume of 100 μL and a bacterial concentration of ~5 × 10^5^ CFU mL^−1^. The microplates were kept in an incubator at 37 °C for 24 h under constant shaking of 100 rpm. The MIC values were taken as the concentration of the mesoporous particles at which no microbial growth was visually observed. A positive control without the mesoporous silica and a negative control without bacteria were also prepared. All the tests were run in triplicate.

#### 2.11.2. Antimicrobial Assessment of Composites Prepared Using Hybrid MCM-41@Ag Particles

Antimicrobial tests of the prepared composite films based on PLLA with several amounts of mesoporous silica with and without Ag modification were performed following the E2149-13a standard method from the American Society for Testing and Materials (ASTM) [33], which is a standardized method to evaluate the antimicrobial activity of material surfaces. Different kinds of bacteria, previously described in the Materials and chemicals section, were used in this analysis. The bacterial suspension was prepared as in a MIC determination process but in PBS at a concentration of ~10^6^ CFU mL^−1^. Composite films were cut into round pieces of 1 cm^2^ and 50–60 mg weight. Each sample was placed in a sterile falcon tube containing 1 mL of the bacterial suspension inoculum and 9 mL of PBS to obtain a working concentration of ~10^5^ CFU mL^−1^. Control experiments were also performed on composite with several amounts of mesoporous not modified with Ag and also blank experiments with only the inoculum in the absence of film. Subsequently, the suspensions were shaken at 120 rpm for 24 h at 37 °C. After this time, 1 mL of each solution was taken and 1:10 serially diluted. Then, 1 mL of the dilutions was placed on 5% sheep blood Columbia agar plates and incubated for 24 h at 37 °C. In order to determine the number of microorganisms in each sample, just a plate count method was used. The measurements were made at least in triplicate.

## 3. Results

### 3.1. Characteristics of the Hybrid MCM-41@Ag Particles

**Figure 1:**
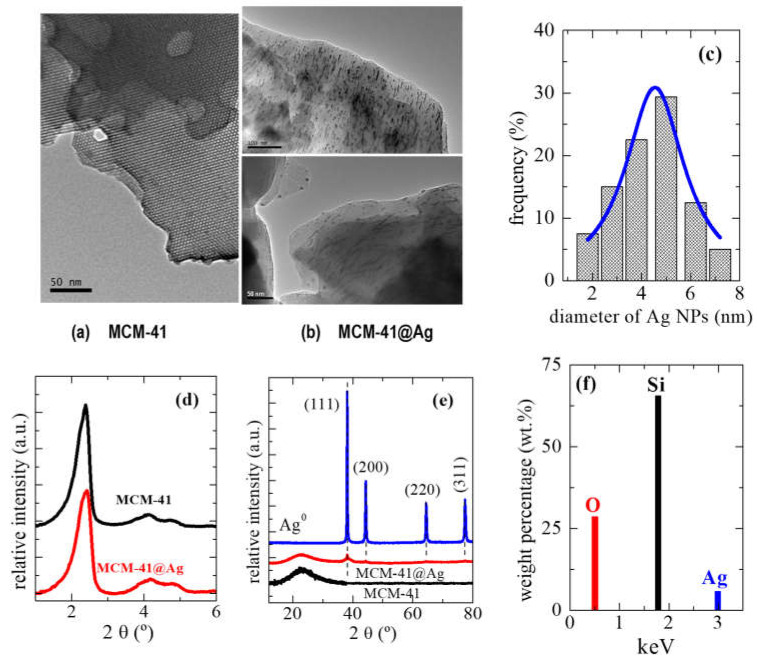
TEM images for (**a**) MCM-41 and (**b**) MCM-41@Ag at two magnifications: scale bar of 100 nm (**top**) and of 50 nm (**bottom**). (**c**) Histogram of the average diameter of the Ag nanoparticles (over 160 units). (**d**) X-ray patterns at middle angles for pristine MCM-41 and functionalized MCM-41@Ag. (**e**) WAXD profiles of MCM-41, MCM-41@Ag, and metallic Ag. (**f**) Energy levels correspond to the three elements present in the hybrid MCM-41@Ag particles (O, Si, and Ag) and their weight percentage.

Figure 1 summarizes the most important features found in the pristine MCM-41 and in the mesoporous particles decorated with Ag. Figure 1a shows the characteristic channels packed in a well-ordered hexagonal arrangement found in the neat MCM-41. This well-organized frame is maintained after the functionalization of MCM-41 with Ag, as seen in Figure 1b. Moreover, the X-ray profile of MCM-41@Ag, represented in Figure 1c, also shows the three main diffraction peaks of its hexagonal morphology, indexed as (100), (110), and (200) reflections in order of increasing angles, similar to the diffractogram of pristine MCM-41.

The incorporation of metallic Ag has taken place mainly as short and uniformly distributed nanowires, as deduced from both images in Figure 1b, although a small fraction of Ag nanoparticles is also noticed. The nanowires exhibit an average length of 40 nm, while the mean diameter in the nanoparticles is around 4.5 nm, as deduced from the histogram represented in Figure 1c.

Formation of metal or semiconductor nanowires within mesoporous MCM-41 materials is not an easy task, and in many cases, nanoparticles are achieved [34]. Here, a simple chemical methodology described for the formation of Ag nanowires within mesoporous SBA-15 has been used [25], consisting basically of the impregnation of mesoporous particles within an AgNO_3_ solution followed by thermal decomposition at high temperature in order to boost the Ag reduction. The incorporation of Ag^0^ within the MCM-41 can be deduced from Figure 1d through the observation of its characteristic diffractions at the wide-angle range, which is superimposed to the amorphous halo common in the mesoporous silica. The Ag content in this hybrid silica can be determined from its WAXD profile through a comparison of areas of the (111) reflection in the Ag^0^ and MCM-41@Ag after their respective normalization. Thus, a content in Ag of 5.9 ± 0.2 wt.% is achieved, which is in very good agreement with the composition estimated by energy dispersive X-ray (EDX) spectroscopy, as noticed in Figure 1f. Accordingly, these hybrid MCM-41@Ag particles can be considered as short Ag nanowires encapsulated within the mesoporous MCM-41 channels, a fact that might contribute to preserving the Ag antimicrobial characteristics because Ag aggregation is minimized.

The biocidal response to different microorganisms was checked in these hybrid MCM-41@Ag particles. The minimum inhibitory concentration (MIC) obtained, which provides an idea of the activity of a given antimicrobial agent against a particular microorganism, is listed in Table 1.

The neat MCM-41 particles do not exhibit any inhibitory response, as expected. When a 5.9 wt.% of Ag is added as short nanowires, the lowest value of MIC is found for *C. parapsilosis*, while the highest is presented by *E. coli*, meaning that the worse antimicrobial reduction is shown against this *E. coli* bacteria. All of these values show a very satisfactory antimicrobial inhibitory ability, taking into consideration the low content of silver within the hybrid MCM-41@Ag particles. Furthermore, the MIC values have been recalculated for the actual amount of Ag, and an improvement in effectiveness is noticed [35,36]. Once hybrid MCM-41@Ag particles are incorporated into PLLA, evaluation of behavior against microorganisms in the resultant composites will enable knowing if this initial adequate biocidal response is or is not transferred to the final materials and if their response against microbes is sufficiently good. This point will be discussed later on.

### 3.2. Morphological Details of Composites

**Figure 2:**
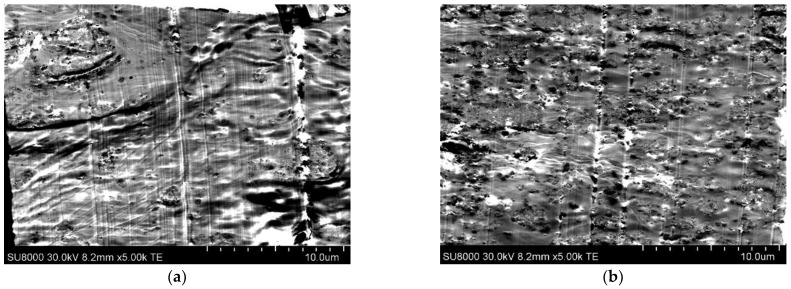
FESEM micrographs for PLLAMCM@Ag9 (**a**) and PLLAMCM@Ag20 (**b**) composites.

Figure 2 displays FESEM images for some of the PLLA composites incorporating hybrid MCM-41@Ag particles. Both pictures allow observation that the modified MCM-41@Ag silica is rather well dispersed within the PLLA matrix for the PLLAMCM@Ag9 and PLLAMCM@Ag20 composites. In addition to this uniform distribution, it has to be commented that the formation of aggregates of large size is not observed. These features indicate that the protocol followed during the preparation of composites leads to rather satisfactory results.

Although films are finally attained by melt compression in the latest stage to avoid the presence of a residual solvent, the incorporation of the hybrid mesoporous silica into the PLLA was performed by casting. An identical route was applied in the materials containing neat mesoporous silica, turning out also a suitable distribution of those pure particles within the polymeric matrix.

### 3.3. Thermal Stability of the Different Composites

**Figure 3:**
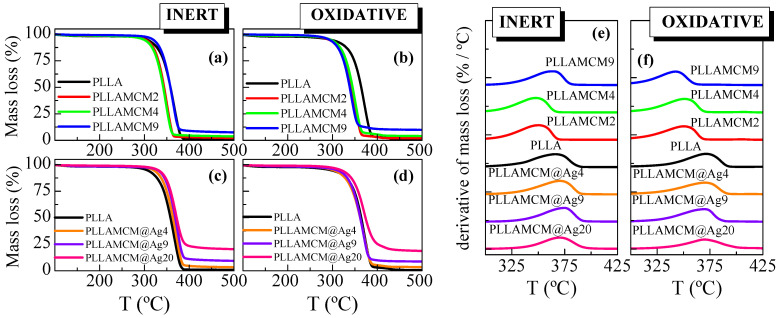
TGA curves for PLLA composites with pristine MCM-41 or decorated with Ag particles under nitrogen: (**a**,**c**) graphs, respectively, and under air: (**b**,**d**) representations, respectively. Amplified DTGA curves under inert and oxidative atmospheres: (**e**,**f**) representations.

Thermogravimetric analysis (TGA) has been carried out for all of the PLLA composites. Figure 3 shows the curves under inert and air atmospheres (on the left part, representations from (a) to (d)) and their corresponding derivatives (on the right, representations from (e) to (f)). Two different trends are observed depending on the type of MCM-41 particles used: pristine or functionalized. Inert decomposition, independently of analyzing pure PLLA or its compounds with neat MCM-41 and with the particles decorated with Ag, takes place through a unique stage. However, the incorporation of small amounts of pristine MCM-41 (PLLAMCM2 and PLLAMCM4 specimens) leads to a significant reduction in thermal stability in an inert medium of this PLLA (with a content in the *L* isomer of 2 mol%). Thus, the maximum estimated from DTGA curves is found in PLLA at 365.5 °C, this being shifted to 349.5 °C and to 347.0 °C for the PLLAMCM2 and PLLAMCM4 samples, respectively. The decrease is smaller in the PLLAMCM9 composite, appearing at 363.5 °C. This effect has already been described in works of literature. In fact, neat mesoporous silicas are used, sometimes to act as catalysts during polymer decomposition. The effectiveness of MCM-41 was previously described in polyethylene degradation under an inert atmosphere [37,38]. This character of boosting degradation was also observed for in situ polymerized polyethylene-based composites when MCM-41 particles, neat and modified with either undecenoic acid or silanes, were employed in a double role, as catalyst support and as fillers [39,40,41].

Composites containing Ag nanowires within the mesoporous MCM-41 channels (PLLAMCM@Ag4, PLLAMCM@Ag9, and PLLAMCM@Ag20 samples) show, however, a thermal stability slightly greater than pure PLLA since their maxima are located, independently of functionalized silica composition, at around 371 °C in the DTGA curves. Thus, the presence of Ag seems to contribute to an improvement in the thermal stability of the resulting materials.

Two degradation mechanisms are exhibited in the TGA curves under air for PLLA. The main decomposition stage, which occurs at the lowest temperature, involves most of the mass loss, while a secondary process takes place immediately when the major process finishes. Again, a dual trend is observed when measurements are performed under an oxidative environment depending on the type of MCM-41 particles added. The composites with neat MCM-41 exhibit lower thermal stability than pure PLLA, as deduced from Figure 3b,f, while the maximum temperature of PLLA decomposition is maintained rather constant in those incorporating hybrid MCM-41@Ag, as seen in Figure 3d,f.

Furthermore, the specific amount of the inorganic loading (either pristine MCM-41 or decorated with Ag particles) incorporated during the preparation is assessed from these curves achieved under the two environments. The values calculated are fairly analogous, pointing out indirectly the homogeneity in the distribution of the inorganic particles within the PLLA matrix. The average data (listed in Table 2) are those used in the name of the different composites.

### 3.4. Thermal Transitions of the Composites

**Figure 4:**
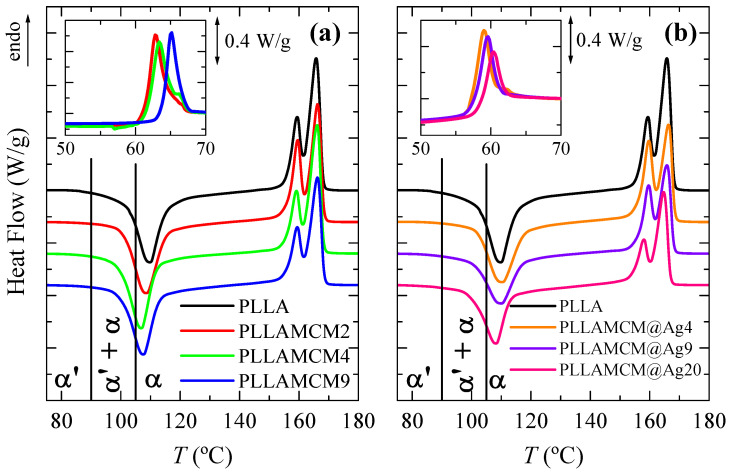
DSC heating curves (endo up) were carried out at 10 °C/min for the PLLA analyzed and its composites prepared using pristine MCM-41 (**a**) and hybrid MCM-41@Ag (**b**) particles.

Figure 4 displays the thermal transitions on the first heating of the pure PLLA and the several composites. Films from all of them have been manufactured by compression molding at a relatively fast cooling rate, a fact leading to completely amorphous materials. Consequently, a cold crystallization is observed above glass transitions, followed by the melting process of those cold-crystallized entities, which is composed of double endothermic peaks, as observed in all of the specimens independently of the absence (neat PLLA) or presence of silica and if the mesoporous particles are or not decorated with Ag. These two melting peaks are attributed to melting-recrystallization processes.

Another characteristic that is clearly noticeable is the nucleating effect that pristine or Ag functionalized silica plays on the PLLA crystallization. Accordingly, the value of cold crystallization temperature (T_c_^cold^) at minimum is 109.5 °C for the PLLA matrix and 108.5 °C, 107 °C and 107.5 °C in PLLAMCM2, PLLAMCM4, and PLLAMCM9 samples, respectively, while reaching 110 °C, 109 °C and 108 °C, respectively for the PLLAMCM@Ag4, PLLAMCM@Ag9, and PLLAMCM@Ag20 specimens (see data in Table 2). If materials with similar loading composition are compared, i.e., PLLAMCM4 with PLLAMCM@Ag4, and PLLAMCM9 with PLLAMCM@Ag9, the nucleating ability of the hybrid particles is reduced in comparison with that displayed by the neat silica. Another difference is found in the glass transition (T_g_) region. In the composites containing pure mesoporous particles, the T_g_ is located at 58 °C for the PLLA and at 60 °C, at 61 °C and at 63 °C in PLLAMCM2, PLLAMCM4 and PLLAMCM9, respectively. Furthermore, they exhibit the common enthalpic relaxation phenomena, as seen in the inset of Figure 4a. Composites prepared by hybrid MCM-41@Ag particles show values of T_g_ rather constant and similar to that corresponding to PLLA, as depicted in the inset of Figure 4b. Concerning crystalline lattice formed in the samples along cold crystallization, the α form is expected to be developed in the majority since this PLLA generates this polymorph above 105 °C [12], and all these samples show the cold crystallization at higher temperatures. Consequently, the characteristic α′→α transition [17,18,28] is not observed in any case.

**Figure 5:**
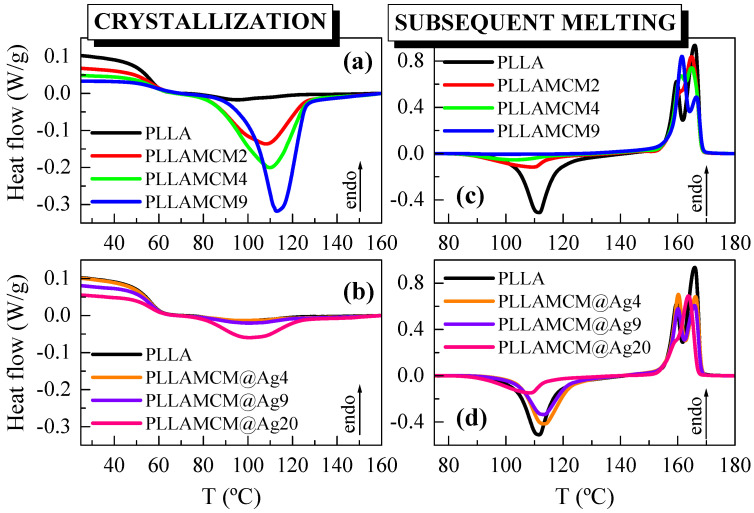
DSC cooling curves carried out in experiments at 10 °C/min for the different composites: (**a**) neat MCM-41 or (**b**) hybrid MCM-41@Ag particles, and the subsequent heating curves at 10 °C/min for composites with (**c**) neat MCM-41 or (**d**) hybrid MCM-41@Ag silicas.

Differences in the PLLA transitions between the composites incorporating pristine or modified MCM-41 become larger if crystallization at 10 °C/min and the subsequent melting are analyzed, as observed from Figure 5a,b. By focusing the attention on the former, it can be deduced the important nucleating influence that the incorporation of pristine MCM-41 exerts in the PLLA crystallization on cooling at that rate. Thus, pure PLLA crystallized in a very small amount, appearing at its peak minimum (T_c_) at 95.5 °C, while the area of this exothermic event noticeably increases, and its location is moved to considerably lower temperatures as MCM-41 content is enlarged (see values in Table 2). In this context, talc is considered an effective nucleating agent for PLLA and frequently is used as a reference [42]. Peak crystallization temperature was described to shift from 107 °C to 123 °C when 3% talc was incorporated via solution blending in experiments using a cooling rate of 1 °C/min [43]. The present results with pristine MCM-41 are even better, taking into account that the cooling rate is here much faster. Functionalization of MCM-41 with short Ag nanowires within its mesostructure also favors PLLA crystallization, although to a much smaller extent, as seen in Figure 5b. Therefore, the T_c_ shift is less important in these composites. In fact, T_c_ in the PLLAMCM@Ag20 sample is displaced up to 101 °C.

These characteristics found during cooling also affect the behavior along the subsequent heating scan. On the one hand, cold crystallization is minimized in the materials containing neat MCM-41 silica, this exothermic process being completely absent in PLLAMCM9 since the whole crystallization has occurred on cooling at 10 °C/min. In the PLLAMCM@Ag materials, cold crystallization also becomes less significant compared with pure PLLA since part of their PLLA chains crystallized during cooling, although hybrid MCM@Ag particles turn out less effective. As in the first heating scan, the melting process is composed of double endothermic peaks, which are ascribed to melting-recrystallization events.

### 3.5. Crystalline Features in the Different Composites

Crystallization in all of the materials analyzed, either on heating or on cooling experiments, takes place in a temperature interval at which the α polymorph is expected to be developed in majority according to the characteristics previously described for this particular PLLA [12] and to the absence of the distinctive DSC transition, where the α′ crystals melt and, then, the α ones are crystallized [17,18,28]. This assumption has been ascertained by variable-temperature WAXS experiments, performed only in the composites incorporating hybrid MCM-41@Ag particles, results being extrapolated to those materials prepared from neat MCM-41 particles. Since crystallization range is similar between these two families despite the fact that the nucleating effect of the pristine silica is much more considerable than that found for the hybrid MCM-41@Ag particles.

**Figure 6:**
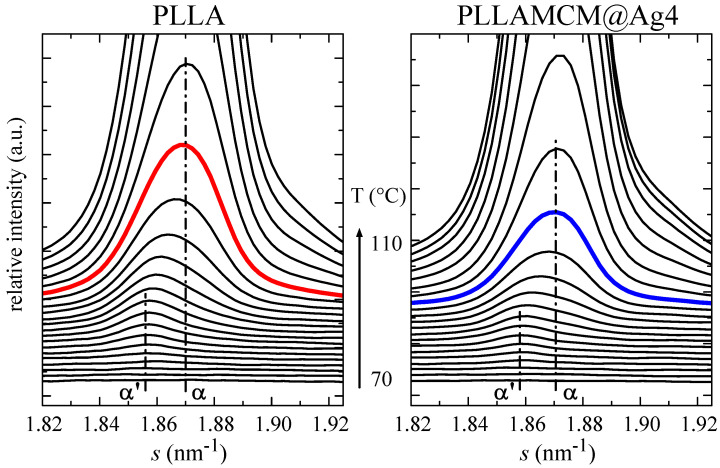
Dependence on the temperature of PLLA and PLLAMCM@Ag4 diffractograms in the scattering vector interval related to the pure (110)/(200) crystal reflection obtained from synchrotron WAXS experiments on heating at 10 °C/min. Red and blue profiles indicate the temperature up to which maximum is progressively moved to higher *s* values.

The most common PLLA polymorphs, α′ (also designated, sometimes, as δ [44]) and α lattices, show very analogous X-ray profiles, only varying slightly the location of their most intense diffractions, the (110)/(200) and the (203) ones, and by the appearance of some other specific weak reflections [17,45,46]. Thus, in order to easily notice the position of diffractions from both α′ and α crystalline modifications, dependence on temperature of only the major (110)/(200) reflection, after subtraction of the amorphous halo [6], is represented in Figure 6 for the pure PLLA and the PLLAMCM@Ag4. These two samples, similarly to the rest of the materials studied, are completely amorphous after their processing by melt compression, as deduced from DSC results attained during the first heating scan. Thus, the profile at 70 °C in Figure 6 is a near-zero line because the PLLA amorphous halo has been subtracted [6], and the crystallinity is negligible. As temperature increases, a small reflection appears in this *s* range. Its initial location, compared with that described in the literature [47], along with its shift to higher values with growing temperatures, seems to indicate that the α′ form has crystallized at first. The nucleating effect of the hybrid MCM-41@Ag particles is noticed, and the crystalline phase is observed in the PLLAMCM@Ag4 composite at temperatures slightly lower than in the PLLA matrix. As the temperature is further raised, the intensity is enlarged in both cases, and the location of its maximum is progressively moved to higher *s* values; this shift is observed until 102 °C in PLLA (highlighted in red) and until 98 °C in PLLAMCM@Ag4 (highlighted in blue). After that, the position is maintained almost constant, and only its intensity significantly increases.

**Figure 7:**
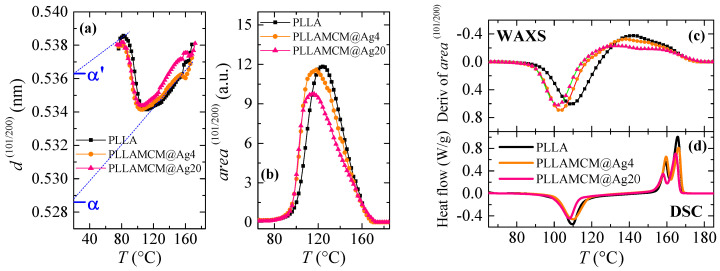
Dependence on temperature, in melting experiments at 10 °C/min, of (**a**) spacing, (**b**) area, and (**c**) derivative of the area for the main (110/200) diffraction in PLLA, PLLAMCM@Ag4, and PLLAMCM@Ag20. For comparison, the corresponding DSC melting curves are also shown (**d**). The thick marks labeled as α′ and α in the plot (**a**), are the values described at room temperature in ref. [47] for both modifications.

This gradual shifting to higher *s* values until around 100 °C and, consequently, to smaller spacings, as noted in Figure 7a (including also the results from PLLAMCM@Ag20), corresponds to the transition from that α′ polymorph to the α form [6]. Formation of the α′ modification is only developed in a minor amount, and the α phase is the majority crystalline structure. Despite the similarity of X-ray patterns for both polymorphs, synchrotron measurements are sensitive to the α′ development and its transition to the α form through the variation of spacings in this main (110/200) reflection, although this transition was not evident by DSC because of the small amount of α′ crystallites involved. On the other hand, dependence on the temperature of the area of this diffraction and of its derivative (Figure 7b,c) agrees very well with results obtained from DSC with respect to the cold crystallization and the melting processes, but the transformation from α′ to α modifications is not evident [6] from these parameters.

**Figure 8:**
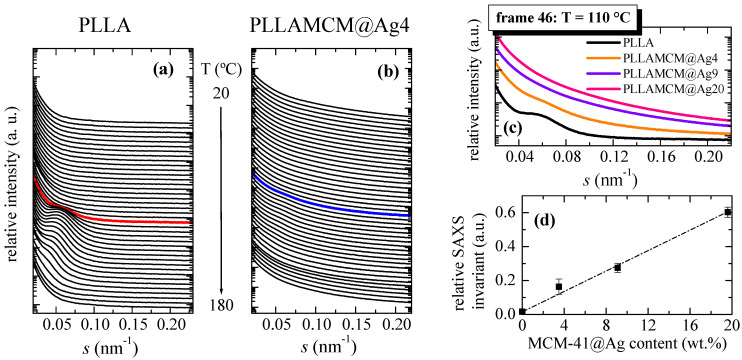
SAXS profiles during melting experiments at 10 °C/min for the pure PLLA (**a**) and the PLLAMCM@Ag4 composite (**b**). SAXS frames at 110 °C for PLLA and all of the materials incorporating MCM-41@Ag (**c**). Dependence of relative SAXS invariant on the content in hybrid silica (**d**).

Figure 8a,b show variations of the synchrotron SAXS 1D profiles with temperature for PLLA and PLLAMCM@Ag4. Long spacing, L, could be determined from these curves when PLLA crystallizes either in the pure polymer or acting as a matrix in the composites. Therefore, no peak for estimation of long spacing is detected in the samples just processed by compression molding since they are completely amorphous, as confirmed by DSC and WAXS measurements. As temperature increases, a clear peak is observed in the pure PLLA at about 108 °C while this is noticeably overlapped with the direct beam in the composite and fully merged at higher filler contents, as depicted in Figure 8c. In the literature, the absence of this characteristic peak has been described in samples that mainly show the α′ polymorph, except when this crystalline structure is rather perfect [6]. The small variations existing between the α′ and α-crystallites, which are related to chain conformation and packing mode, imply a greater lateral disorder that avoids the appearance of long spacing. This parameter provides valuable data in the lamellar stack model theory for semicrystalline polymers. Long spacing deals with electron density changes between the two amorphous and crystalline phases so that it displays rather significant differences during crystallization, recrystallization, or melting processes in semicrystalline polymers [48,49,50], triggering considerable structural information.

Figure 8c displays the results observed at a temperature of 110 °C when the α phase has already developed significantly (see Figure 7a,b) in PLLA and in its composites with MCM-41@Ag particles. The long spacing peak is clearly seen in the pure polymer and appears as a small shoulder in PLLAMCM@Ag4, but it is not distinguished in PLLAMCM@Ag9 and PLLAMCM@Ag20, as aforementioned. Simultaneously, the area under the curve, i.e., the invariant, increases considerably as content in hybrid particles does. In fact, the rise of the relative SAXS invariant is rather linear with the amount of decorated mesoporous MCM-41@Ag silica, as noticed in Figure 8d, with the effect of masking the long spacing for the higher Ag contents. The disappearance of the peak ascribed to long spacing might be related to partial radiation shielding triggered by the presence of Ag^0^ in these composites. This is a quite common feature when metallic particles are incorporated into polymers [51,52].

### 3.6. Mechanical Behavior of the Different Composites

**Figure 9:**
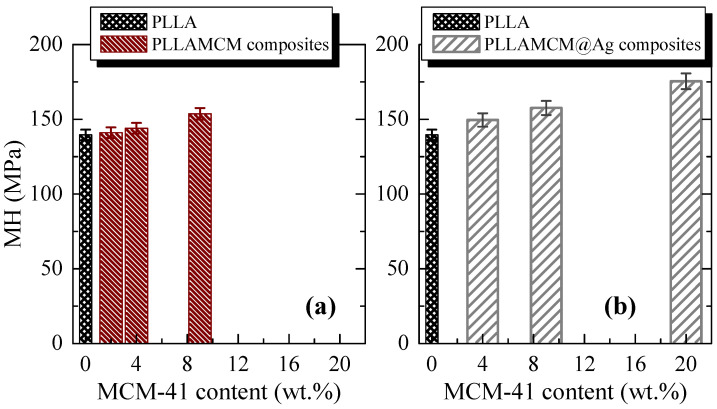
Microhardness, MH, dependence on mesoporous silica content for the different composites: (**a**) those prepared using neat MCM-41 silica; (**b**) the ones obtained incorporating hybrid MCM-41@Ag particles.

Any material intended to have practical applications must display suitable mechanical parameters independently of other particular and specific characteristics. Thus, a minimum mechanical performance enables its final applicability. In order to check how the incorporation of either neat MCM-41 or their hybrid particles with Ag affects the PLLA response, microhardness (MH) measurements have been carried out. The results are represented in Figure 9. The experiments are performed on the initial films, i.e., those prepared by compression molding, which is completely amorphous (see data in Table 2 and Figure 6). In this manner, differences related to crystalline characteristics focused mainly on their distinct nucleating capacity or on variation in the degree of crystallinity, are eluded when these two types of particles are incorporated into PLLA.

Pure PLLA is the softest material when compared with the different composites. In the two sets of composites, a growth in MH is observed as increasing particle content, as expected. This parameter is related to stiffness [30,31,53], and either pristine or decorated silicas are a component harder than the polymeric matrix. Concerning the nature of the loadings, slightly higher MH values are found in the family containing the hybrid MCM-41@Ag silica compared with those achieved by adding the pristine MCM-41. In fact, PLLAMCM4 and PLLAMCM9 show an MH of 144 and 154 MPa, respectively, while 158 and 175 MPa are the values for PLLAMCM@Ag4 and PLLAMCM@Ag9, respectively. This small difference might be associated with the presence of a small amount of Ag^0^ in the latest materials, which provides an extra reinforcement.

### 3.7. Antimicrobial Efficacy of Composites with Hybrid MCM-41@Ag Particles

Several microorganisms have been selected for analyzing the antimicrobial efficiency of pure biobased and biodegradable PLLA and its composites with functionalized MCM-41 particles, where short nanowires of Ag^0^ were grown: *S. epidermidis* and *S. aureus* as Gram-positive bacteria; *P. aeruginosa* and *E. coli* as Gram-negative ones; and the *C. parapsilosis* as yeast. This antimicrobial activity is studied using a method from the American Society for Testing and Materials (ASTM) against Gram-positive and Gram-negative bacteria and yeast, using as controls: microorganisms without material, the neat PLLA, and the PLLAMCM9 composite with pristine MCM-41 silica. Data in Table 3 detail that these two, PLLA and PLLAMCM9, do not display any biocide effectiveness, as expected. Incorporation of a small amount of hybrid MCM-41@Ag particles in the PLLAMCM@Ag4 composite, with only content in Ag of around 0.25 wt.%, leads to the appearance of a small activity, exclusively against *S. epidermidis* and *C. parapsilosis.* A further increase in the number of modified MCM-41 particles and, thus, of a greater content in nanowires of Ag^0^ (up to about 0.55 wt.%) significantly improves the activity against *S. epidermidis* maintains that against *C. parapsilosis* and now provides biocidal efficiency against *S. aureus, P. aeruginosa,* and *E. coli.*

A larger content (around 1.2 wt.%) of the short Ag^0^ nanowires existing in the PLLAMCM@Ag20 material leads to a very remarkable antimicrobial efficiency, enabling a reduction higher than the 97% of all the different microorganism colonies, then presenting broad-spectrum antimicrobial activity. In the literature, it has been described that the antibacterial efficacies after 12 h against *S. aureus* and *E. coli* were 98.5% and 94.2%, respectively, for Ag/PLA fibers prepared by electrospinning (with an AgNO_3_ content by weight of 32 wt.% in the spinning solution with respect to PLA) [54], while the highest reduction in viability after 2 h found in 3D printed PLA/AgNP nanocomposites, with content in Ag NPs of 9.4 wt.%, was 74.9 against *S. aureus* and 94.9 for *E. coli* [55]. Comparison of these results with the ones reported here (being cautious because sometimes such comparisons do not turn out completely accurate due to differences in the determination method employed or in the bacteria strains used) proves that the biocidal response of PLLAMCM@Ag9 and PLLAMCM@Ag20 against these two microorganisms is much better (see values in Table 3) considering that the Ag content is 0.55 wt.% for the former and 1.2 wt.% in the latter, respectively. Keeping in mind this small content in Ag^0^, the PLLAMCM@Ag20 material is rather active against *P. aeruginosa*, the bacteria that most often causes infections in humans. Infections caused by *P. aeruginosa* may include but are not limited to those in broken or ulcerated skin, in blood, in a heart valve, or in lung infections.

PLLA can be easily processed by 3D printing, a technique that is currently being explored for heart valve manufacturing [56]. This technology could allow any device to be customized for each patient, thus becoming very important in the medical sector. In addition, the use of an appropriate biomaterial (the three components of these hybrids are approved by the FDA) with antimicrobial activity could minimize infection probability in these heart valves during their service life, and replacement of the valves could be extended from above 10 years (which is their average life shelf). Concerning skin infections, another common bacterium is the *S. epidermidis*, in addition to the *P. aeruginosa.* Thus, the use of dressings containing antibacterial substances can avoid the need to consume oral antibiotics if the infection is not very severe, allowing the complete restoration of the skin.

Ag NPs are quite an effective antimicrobial as it has been shown to exert high activity against both Gram-positive and Gram-negative bacteria [57,58]. In addition, several studies reported that the growth of yeast was considerably weakened when spores were incubated in direct contact with Ag NPs [59,60]. This kind of nanoparticle interacts with thiol groups in proteins resulting in the inactivation of respiratory enzymes and leading to the production of ROS (reactive oxygen species). It was also shown that Ag^+^ ions prevent DNA and RNA replication and affect the structure and permeability of the cell membrane [61]. The release rate of Ag^+^ ions depends in part on the size of Ag NPs: those with smaller sizes might induce faster Ag^+^ ion liberation [62]. The complex environment of culture and the dead bacteria often cause the aggregation of Ag NPs. Their resultant larger size reduces the release of Ag^+^ ions, leading to the decrease or loss of antibacterial activity [35].

The good results obtained here can be related, on the one hand, to the shape and size of the Ag since it is mostly found as small nanosized wires and not as nanoparticles (which can also be seen, although in the minority) that are located within the MCM-41 mesostructure. The nanowire size is small since they are short in length and their diameter is inferior to the size of the MCM-41 hexagonal arrangement, i.e., around 3 nm. On the other hand, the presence of those nanowires within the MCM-41 particles avoids Ag aggregation and decomposition, and the release of Ag^+^ from a porous network is facilitated [39] features that could enable long-term antimicrobial release. These results are remarkable not only because a small amount of Ag shows a suitable capacity for a microbial reduction but also because, in these experiments, Ag is not in solution: Ag is confined in MCM-41 particles that are, in turn, embedded in a PLLA matrix. i.e., in solid materials, meaning that Ag inhibitory capacity for the elimination of microbes is transferred to the bulk in the final PLLA composites.

## 4. Conclusions

Several composites are attained based on PLLA and either pure MCM-41 mesoporous silica or hybrid MCM-41 decorated with Ag (MCM-41@Ag) by a solution/evaporation protocol, being the final films processed by compression molding in a subsequent stage. In these composites, the modified MCM-41@Ag particles are quite well dispersed within the PLLA matrix, and the formation of agglomerates of large size is not detected. Thus, the protocol followed during preparation leads to rather satisfactory morphological aspects. In addition, the approach selected for the functionalization of MCM-41 with Ag allows for achieving Ag nanowires within its unfilled channels and Ag nanoparticles on the silica surface. In the composites, the PLLA thermal stability, nucleation efficiency, and mechanical response are mainly dependent on the MCM-41 nature (functionalized or not). Moreover, real-time variable-temperature measurements using synchrotron radiation indicate that the disordered α′ polymorph is initially formed during cold crystallization, but it rapidly transforms into ordered α crystals. Since PLLA is the softest material, a growth in MH is found to increase silica content, with slightly higher MH values in the composites containing the hybrid MCM-41@Ag silica. Finally, a remarkable antimicrobial activity against several microorganisms (Gram-positive and Gram-negative bacteria and yeast) has been found in the PLLA composites with MCM-41@Ag, being particularly active against the *S. epidermidis* and *P. aeruginosa*. Thus, the Ag inhibitory capacity for the elimination of microbes is transferred to the bulk in the final PLLA composites. These outstanding results seem to be related to the appropriate shape and size of the Ag in the composites (with Ag confined in the channels of MCM-41 particles), even though the amount of Ag added is very small.

## Figures and Tables

**Table 1:** Values of minimum inhibitory concentration (MIC) for several microorganisms found in neat and Ag-functionalized MCM-41 particles.

Sample	Ag Content (wt.%)	MIC (µg/mL)				
		*S. aureus*	*S. epidermidis*	*E. coli*	*P. aeruginosa*	*C. parapsilosis*
MCM-41	0	>500	>500	>500	>500	>500
MCM-41@Ag	5.9	62.5	32	125	62.5	7.8
Ag *	100	3.8	1.9	7.5	3.8	0.5

* Values recalculated for the actual amount of Ag nanowires within the MCM-41 mesostructure.

**Table 2:** DSC results for the different samples: cold crystallization temperature (T_c1_^cold^ and T_c2_^cold^)–estimated from the first and second heating runs, respectively–; first and second melting temperatures (T_m1_^1^, T_m1_^2^, T_m2_^1^, and T_m2_^2^)–estimated from the first and second heating runs, respectively–; crystallization temperature(T_c_^C^); and overall crystallinity (normalized to the actual PLLA content in the material) for the first heating run (f_c_^1R^), cooling (f_c_^C^) as well as for the cold crystallization (f_c2_^cold^) and melting (f_c_^2R^) processes of the second heating run.

Sample	Silica Content (wt.%)	T_c1_^cold^ (°C)	T_m1_^1^ (°C)	T_m1_^2^ (°C)	f_c_^1R^	T_c_^C^ (°C)	f_c_^C^	T_c2_^cold^ (°C)	f_c2_^cold^	T_m2_^1^ (°C)	T_m2_^2^ (°C)	f_c_^2R^
PLLA	0	109.5	159.5	166.0	0.0	95.5	0.04	111.0	0.42	159.5	166.0	0.03
PLLAMCM2	2.1	108.5	159.5	166.0	0.0	108.0	0.27	109.5	0.16	160.5	164.5	0.27
PLLAMCM4	3.9	107.0	159.5	166.0	0.0	110.0	0.37	102.5	0.08	161.0	165.0	0.37
PLLAMCM9	8.7	107.5	159.5	166.0	0.0	114.0	0.48	98.0	0.01	161.5	166.5	0.47
PLLAMCM@Ag4	3.5	110.0	159.5	166.0	0.0	98.0	0.04	113.0	0.38	160.0	166.0	0.02
PLLAMCM@Ag9	9.1	109.0	159.5	166.0	0.0	100.0	0.06	113.0	0.34	160.0	166.0	0.04
PLLAMCM@Ag20	19.6	108.0	158.0	165.0	0.0	101.0	0.17	108.0	0.20	159.0	164.0	0.15

**Table 3:** Antimicrobial activity expressed as a percentage of microorganism reduction (%) of composites with and without Ag against *S. epidermidis*, *S. aureus*, *P. aeruginosa*, *E. coli,* and *C. parapsilosis*.

Sample	Microbial Reduction (%)				
	*S. aureus*	*S. epidermidis*	*E. coli*	*P. aeruginosa*	*C. parapsilosis*
PLLA	-	-	-	-	-
PLLAMCM9	-	-	-	-	-
PLLAMCM@Ag4	66.7	-	-	-	71.4
PLLAMCM@Ag9	99.2	83.3	98.3	77.8	71.4
PLLAMCM@Ag20	99.9	98.7	99.7	98.4	97.1

## Data Availability

Data are contained within the article.
